# Electrochemical Characterization of a Complex FeFe Hydrogenase, the Electron-Bifurcating Hnd From *Desulfovibrio fructosovorans*

**DOI:** 10.3389/fchem.2020.573305

**Published:** 2021-01-08

**Authors:** Aurore Jacq-Bailly, Martino Benvenuti, Natalie Payne, Arlette Kpebe, Christina Felbek, Vincent Fourmond, Christophe Léger, Myriam Brugna, Carole Baffert

**Affiliations:** CNRS, Aix Marseille University, BIP, Marseille, France

**Keywords:** direct electrochemistry, FeFe hydrogenase, electron bifurcation, *Desulfovibrio fructosovorans*, inactivation

## Abstract

Hnd, an FeFe hydrogenase from *Desulfovibrio fructosovorans*, is a tetrameric enzyme that can perform flavin-based electron bifurcation. It couples the oxidation of H_2_ to both the exergonic reduction of NAD^+^ and the endergonic reduction of a ferredoxin. We previously showed that Hnd retains activity even when purified aerobically unlike other electron-bifurcating hydrogenases. In this study, we describe the purification of the enzyme under O_2_-free atmosphere and its biochemical and electrochemical characterization. Despite its complexity due to its multimeric composition, Hnd can catalytically and directly exchange electrons with an electrode. We characterized the catalytic and inhibition properties of this electron-bifurcating hydrogenase using protein film electrochemistry of Hnd by purifying Hnd aerobically or anaerobically, then comparing the electrochemical properties of the enzyme purified under the two conditions *via* protein film electrochemistry. Hydrogenases are usually inactivated under oxidizing conditions in the absence of dioxygen and can then be reactivated, to some extent, under reducing conditions. We demonstrate that the kinetics of this high potential inactivation/reactivation for Hnd show original properties: it depends on the enzyme purification conditions and varies with time, suggesting the coexistence and the interconversion of two forms of the enzyme. We also show that Hnd catalytic properties (Km for H_2_, diffusion and reaction at the active site of CO and O_2_) are comparable to those of standard hydrogenases (those which cannot catalyze electron bifurcation). These results suggest that the presence of the additional subunits, needed for electron bifurcation, changes neither the catalytic behavior at the active site, nor the gas diffusion kinetics but induces unusual rates of high potential inactivation/reactivation.

## 1. Introduction

### 1.1. Electron-Bifurcating Enzymes

Oxidoreductase enzymes usually catalyze electron transfer between one electron donor and one electron acceptor. Electron-bifurcating enzymes are part of the oxidoreductase family but catalyze the reaction between two electron donors and one electron acceptor or one electron donor and two electron acceptors. More importantly, the reactions with the two electron donors or the two electron acceptors are thermodynamically coupled, one of the reactions being exergonic and the other endergonic. The global reaction being exergonic, the energetic coupling enables an endergonic reaction to occur (Baymann et al., 2018). The first electron-bifurcating enzyme characterized was the cytochrome *bc*_1_ complex in which the electron-bifurcating site is a quinone (Mitchell, 1975). More recently, electron bifurcating enzymes were described in which the electron-bifurcation site is a flavin (Herrmann et al., 2008). The common feature of quinones and flavins is their two redox transitions, making them 2-electron centers. A variety of enzymes were described to use the electron bifurcation mechanism for catalysis: electron-transferring flavoprotein (Etf), heterodisulfide reductase/hydrogenase (Hdr-Mvh), NADH-dependent ferredoxin: NADP^+^ oxidoreductase (Nfn), and NADH-dependent FeFe-hydrogenase to name but a few (Peters et al., 2016; Buckel and Thauer, 2018). Electron bifurcation is a mechanism that is emerging as essential for the bioenergetic of many organisms, but this mechanism is still poorly understood, in part due to the low number of model enzymes characterized to date.

### 1.2. Electron-Bifurcating Hydrogenases

NADH-dependent electron-bifurcating hydrogenases are multimeric (tri- or tetrameric) enzymes and are classified A3 according to the hydrogenase classification proposed by Greening et al. (2016) and Søndergaard et al. (2016). They are all of FeFe-type and consist of at least one subunit harboring the catalytic H-cluster (the hydrogenase active site, which consists of a [4Fe4S] cluster bound *via* a cysteine to a 2Fe subcluster), a [2Fe2S]-cluster containing subunit and a subunit that contains a flavin (usually FMN) and FeS clusters as well as an NAD binding site. They catalyze the oxidation of H_2_ coupled to the reduction of both NAD^+^ and a ferredoxin with a bifurcation mechanism and/or the reduction of proton coupled to oxidation of NADH and a ferredoxin with a confurcation mechanism. So far, electron-bifurcating hydrogenases from five anaerobic bacteria have been purified and characterized (Schuchmann and Mueller, 2012; Wang et al., 2013; Zheng et al., 2014), including the electron-bifurcating hydrogenase HydABC from *Thermotoga maritima* (Schut and Adams, 2009) and HndABCD from *Desulfovibrio fructosovorans* (Kpebe et al., 2018). They have been tested for either electron bifurcation, or electron confurcation or for both [hydrogenase from *Moorella thermoacetica* (Wang et al., 2013)]. However, no 3D-structure of an electron bifurcating hydrogenase is available, and the electron pathway and the electron bifurcation site in these enzymes are still controversial subjects. Further characterization of electron bifurcating hydrogenases that could be models of this class of enzyme, will increase the understanding of the overall mechanism of electron bifurcation.

### 1.3. Electrochemistry of FeFe Hydrogenases

Electrochemical techniques to study hydrogenases are developed as a complement to biochemical and spectroscopic techniques (Pershad et al., 1999). The first electrochemical characterization of an FeFe hydrogenase was published on the HydA hydrogenase from *Megasphaera elsdenii* (Butt et al., 1997; Caserta et al., 2018). Since then, FeFe hydrogenases from several organisms have been studied using protein film voltammetry to determine their catalytic properties: HydAB from *Desulfovibrio desulfuricans* (Vincent et al., 2005; Parkin et al., 2006; Goldet et al., 2009; Rodríguez-Maciá et al., 2018), HydA from *Clostridium acetobutylicum* (Baffert et al., 2008, 2011, 2012; Goldet et al., 2009; Orain et al., 2015; Kubas et al., 2017), HydA1 from *Chlamydomonas reinhardtii* (Goldet et al., 2009; Stripp et al., 2009; Baffert et al., 2011; Knörzer et al., 2012; Fourmond et al., 2014; Hajj et al., 2014; Orain et al., 2015; Kubas et al., 2017), CpI, CpII, and CpIII from *Clostridium pasteurianum* (Artz et al., 2019), and HydA from *Solobacterium moorei* (Land et al., 2019). All these FeFe hydrogenases are prototypical A1 monomeric or dimeric enzymes (Søndergaard et al., 2016). Different aspects of the catalytic properties of these enzymes are studied by electrochemical methods: affinity for the substrate (K_m_), effect of pH on the catalytic properties, kinetics of inhibition by small molecules (CO, O_2_, S^2−^, formaldehyde), kinetics of oxidative and reductive inactivation, and catalytic bias. Only recently was a multimeric Hydrogen Dependent Carbon Dioxide Reductase (HDCR) from *Acetobacterium woodii* characterized by electrochemistry (Ceccaldi et al., 2017). This hydrogenase is classified A4. It consists of four subunits: the hydrogenase subunit hosting the H-cluster, the formate dehydrogenase subunit hosting the Mo-*bis*PGD cofactor and two subunits containing several FeS clusters. Electrochemical experiments similar to those developed for prototypic hydrogenases were performed. All these experiments on the different hydrogenases and variants (mutations of specific amino acids) give insight into the catalytic mechanism of FeFe hydrogenases and the molecular determinants of the inactivations. The second and last multimeric hydrogenase characterized by direct electrochemistry belongs to the electron-bifurcating enzyme family (classified A3), the hydrogenase HydABC from *Thermotoga maritima* (Chongdar et al., 2020). The enzyme is heterologously produced in *Escherichia coli* and artificially matured with a synthetic diiron cofactor. The isolated hydrogenase catalytic subunit, as well as the complex HydABC, are adsorbed onto an electrode and cyclic voltammograms are recorded at various pH, to show that the isolated hydrogenase subunit and the trimeric complex behave in a similar fashion.

Here, we aim at determining if the catalytic and inhibition properties are influenced by the additional subunits present in NADH-dependent electron-bifurcating hydrogenases or by the condition of purification. We study the electron-bifucating hydrogenase, HndABCD from *Desulfovibrio fructosovorans* (shortened name Hnd), by biochemical and electrochemical methods. Hnd is homologously produced and purified in a fully matured form (Kpebe et al., 2018). Because Hnd is still active when purified aerobically, we determine how the purification conditions (aerobic vs. anaerobic) influence the catalytic behavior of Hnd.

## 2. Materials and Methods

### 2.1. Enzyme Purification

The production and the purification of Hnd hydrogenase from *Desulfovibrio fructosovorans* under aerobic conditions were previously described (Kpebe et al., 2018). The procedure was modified to maintain anaerobic conditions: all steps were performed in a glove-box (Jacomex, [O_2_] ≤ 2 ppm) except the ultra-centrifugation step, for which anaerobiosis is maintained in the tube using an airtight plug. The cell lysis was performed by sonication (10 cycles of 30 s) and the Strep-tagged hydrogenase was purified on a StrepTactin-Superflow (IBA) column (20 mL) according to the manufacturer's instructions. For the comparison of the catalytic properties, the bacterial culture was split in two, and purifications of the Hnd hydrogenase were performed in parallel under both anaerobic and aerobic conditions.

### 2.2. Catalytic Activity Determination

All assays were performed at 30°C and under anaerobic conditions.

#### 2.2.1. H_2_ Oxidizing Activity With Methyl-Viologen (MV)

H_2_ oxidizing activity measurements were performed in anaerobic quartz cuvettes, under a pressure of H_2_ of 1 bar, in 800 μL of a reaction mixture containing 100 mM Tris-HCl pH 8.0, 2 mM dithiothreitol (DTT), and 50 mM methyl-viologen (MV) (Sigma Aldrich) as an artificial electron acceptor. MV reduction was monitored at 604 nm (ε = 13,600 M^−1^·cm^−1^) using a UV-Vis spectrophotometer Lambda 25 (Perkin Elmer), between 10 and 200 ng of purified Hnd were added to the mixture to start the reaction. One unit of hydrogenase activity corresponds to the uptake of 1 μmol of H_2_/min.

#### 2.2.2. H_2_-Production Activity With Methyl-Viologen (MV)

H_2_ production assays were carried out using dithionite-reduced MV (50 mM of MV were reduced with 0.1 M sodium dithionite) as electron donor, in anaerobic 7 mL-serum bottles containing 1 mL of a reaction mixture composed of 100 mM Tris-HCl pH 8.0. The gas phase was 100% N_2_. H_2_ production was measured using gas chromatography (GC) as previously described (Avilan et al., 2018) and the reaction was started by the addition of 0.3–1.1 μg of purified Hnd. One unit of hydrogenase activity corresponds to the production of 1 μmol of H_2_/min.

#### 2.2.3. Electron-Bifurcating Activity for H_2_ Production

Electron-bifurcating (NAD^+^- and Fd-dependent) H_2_-oxidizing activity was assayed as described previously (Kpebe et al., 2018): in anaerobic quartz cuvettes, under 1 bar of H_2_, in 800 μL-mixture containing 100 mM Tris-HCl pH 8.0, 5 μM of FMN, 5 μM of FAD, and 3 mM NAD^+^ in the presence of 20 μM of purified FdxB ferredoxin from *D. fructosovorans*. NAD^+^- and FdxB-reduction were followed simultaneously by recording a full spectrum every 30 s from 300 to 800 nm for 1 h, using a Cary 60 (Varian) in a glovebox. NAD^+^- and FdxB-reduction rates were determined at 340 and 410 nm, respectively using the QSoas software (Fourmond, 2016), an open source program available at www.qsoas.org. The specific activity is given in μmol of NADH/min/mg. The absorption coefficients used were: ε(NADH) = 6,320 M^−1^·cm^−1^, ε(FdxB_ox_410 nm) = 24,000 M^−1^·*cm*^−1^ and ε(FdxB_red_410 nm) = 12,000 M^−1^·*cm*^−1^ (Kpebe et al., 2018).

### 2.3. Electrochemical Techniques

All electrochemical experiments were carried out with the electrochemical set-up and equipment described in reference (Léger et al., 2004) in a glovebox (Jacomex) filled with N_2_. 1 μL of Hnd enzyme solution was mixed with 1 μL of DTT 1M and 8 μL of phosphate buffer pH 7. The final enzyme concentration was 2–7 μM. The enzyme was adsorbed (1 μL of the previous mix) onto a pyrolytic graphite edge electrode (PGE, surface area ≈3 mm^2^) previously polished with an aqueous alumina slurry (1 μm). The electrochemical cell contained a pH 7 phosphate buffer and was continuously flushed with pure H_2_ or with argon. The temperature was regulated to the desired values by circulating water in the double jacket of the cell. For measuring the rates of inhibition by O_2_ or CO, a stock of a buffer saturated by 100% O_2_ or 1% CO in 99% Argon was kept in a capped serum bottle and small aliquots of this solution were injected into the electrochemical cell using gas-tight syringes. The concentrations of O_2_ and CO were calculated using the Henry's law constants: 1.25 mM (atm O_2_)^−1^ and 1 mM (atm CO)^−1^. The change in H_2_ solubility is only about 12% between 10 and 30°C (Wilhelm et al., 1977). This variation induces a difference in the potential of H^+^/H_2_ Nernst couple of 2 mV. With the surface area (around 3 mm^2^) of the electrode, the maximum current (limitation by mass transport) is 100 μA. In the experiments of this study, the H_2_ oxidation current (maximum 2 μA) is not limited by mass transport (or <2%) (Merrouch et al., 2017). The data were analyzed using the QSoas software (Fourmond, 2016). The protein film loss was included in the data analysis of **Figure 4** according to reference (Fourmond et al., 2009). The effect of film loss on cyclic voltammograms is shown in Supplementary Figure 5.

## 3. Results

### 3.1. Biochemical Comparison Between Aerobically and Anaerobically Purified Hnd

We previously reported the biochemical characterization of the Hnd hydrogenase purified under aerobic conditions (Kpebe et al., 2018). Here, we repeated the same characterization but with the enzyme purified under anaerobic conditions. Table 1 summarizes the results obtained with the enzyme purified under the two conditions. The conditions of purification do not have much influence on the properties of Hnd (K_m_ for methyl-viologen, optimal temperature and pH). If we compare the ratio of H_2_ oxidation activity for the two enzymes (anaerobically- and aerobically-purified Hnd) with methyl-viologen and with physiological partners, the ratio is higher when considering the electron bifurcation activity. It should be noted that the activities are lower than those previously reported (Kpebe et al., 2018) because experiments were performed with enzyme purified from cells grown for a longer period (2 months, Covid19 lockdown period) and at lower temperature (20°C), and the enzyme samples were stored 1 week in liquid nitrogen before electron-bifurcating activity measurements. We already observed that the enzyme activity decreases quickly after purification (by a factor of 10) and then stabilizes (Kpebe et al., 2018). However, we checked that only the specific activity changes upon storage and not the other biochemical and catalytic properties (see Supplementary Table 7).

**Table 1:** Comparison of catalytic properties of Hnd purified either under aerobic or anaerobic conditions.

**Purification conditions**	**Km (MV) (in mM)**	**Optimal T (in ^**°**^C)**	**Optimal pH**	**SA (MV) H_**2**_ oxidation (in U/mg of enzyme)**	**SA (MV) H_**2**_ production (in U/mg of enzyme)**	**SA (bifurcation) H_**2**_ oxidation (in U/mg of enzyme)**
Aerobic	15 ± 2^a^	55^a^	8^a^	475 ± 40	64.5 ± 13	0.21 ± 0.12
Anaerobic	13 ± 2	55	8	740 ± 45	53.5 ± 5	0.72 ± 0.3
Ratio (anaerobic/aerobic)				1.56	0.83	3.43

The values are the average of three independent experiments. T, temperature; SA, specific activity.

a *Data from Kpebe et al. (2018)*.

### 3.2. Electrochemical Characterization of Hnd

Hnd purified under the two conditions (aerobic and anaerobic) was characterized using electrochemical methods developed in our laboratory (Sensi et al., 2017; Del Barrio et al., 2018b). As already observed for the electron-bifurcating trimeric hydrogenase from *Thermotoga maritima* (Chongdar et al., 2020), despite its multimeric form, the Hnd hydrogenase can transfer electrons directly to or from an electrode, without the need for redox mediator. However, it not possible to speculate which is the first electron-relay within the enzyme, i.e., what cofactor interacts with the electrode surface and whether it is unique (several entry points could be possible) because structural information is not available for any electron-bifurcating hydrogenase. Because experiments presented in this study were not performed in presence of NAD or ferredoxin, these catalytic properties are more representative of a non-bifurcation reaction. We characterized Hnd purified under aerobic and anaerobic conditions, and compared the catalytic and inactivation kinetic properties.

#### 3.2.1. Oxidative Inactivation and Catalytic Bias

##### 3.2.1.1. Oxidative Inactivation

**Figure 1:**
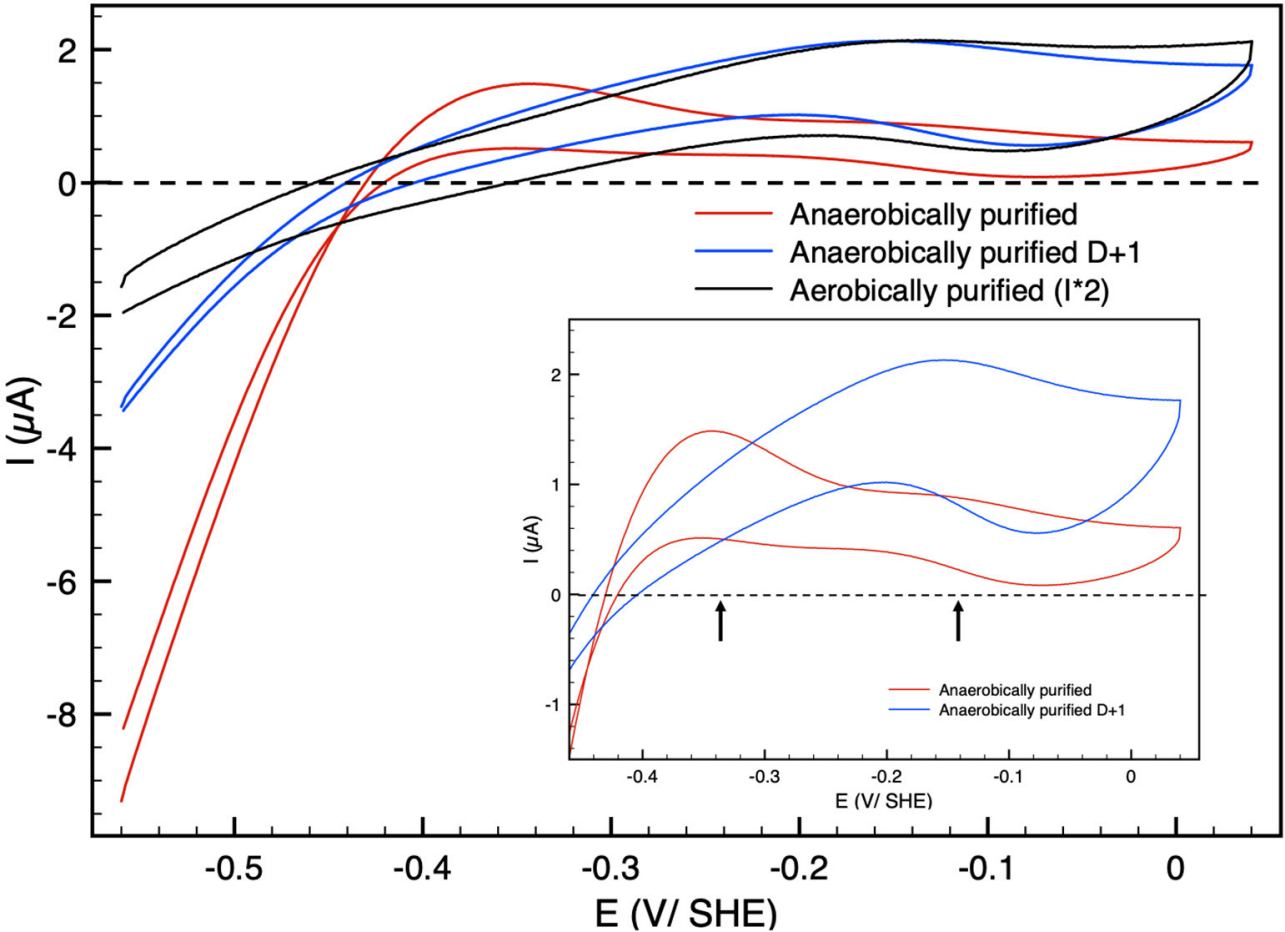
Cyclic voltammograms of Hnd hydrogenase adsorbed on a PGE electrode; black: aerobically-purified enzyme, red: anaerobically-purified enzyme, blue: anaerobically-purified enzyme, stored 1 day (D + 1) anaerobically at 4°C. The current for the aerobically-purified enzyme (black line) was multiplied by two for the sake of clarity. Insert: zoom in the high potential range. Scan rate: 20 mV/s, *T* = 30°C, 1 bar H_2_, phosphate buffer pH 7, ω = 3,000 rpm.

Figure 1 shows catalytic cyclic voltammograms (current as the function of potential) of Hnd adsorbed at a PGE electrode for three enzyme samples: aerobically-purified Hnd, anaerobically-purified Hnd, and anaerobically-purified Hnd stored 1 day at 4°C in a glove-box that contains about 10% dihydrogen. The three voltammograms show a decrease in the catalytic current at high electrode potential, indicating an oxidative inactivation that is reversible as shown on the reverse scan by the increase in current when the potential is decreased. However, the reactivation is not complete, particularly for anaerobically purified Hnd, as shown by the red voltammogram.The irreversible loss could be due to protein film desorption and/or irreversible inactivation. We cannot discriminate between these two processes. It should be noted that at this scan rate, for aerobically-purified Hnd (black line in Figure 1), the decrease in current occurs at around −0.15 V vs. SHE. In the inset of Figure 1, the cyclic voltammogram of the anaerobically-purified Hnd (red line) shows two inactivation/reactivations (indicated by the arrows) at electrode potential around −0.35 V vs. SHE and around −0.15 V vs. SHE. After 1 day of storage of the anaerobically-purified enzyme, the shape of the cyclic voltammogram is similar to that obtained with aerobically-purified Hnd (blue and black lines in Figure 1).

##### 3.2.1.2. Catalytic Bias

**Figure 2:**
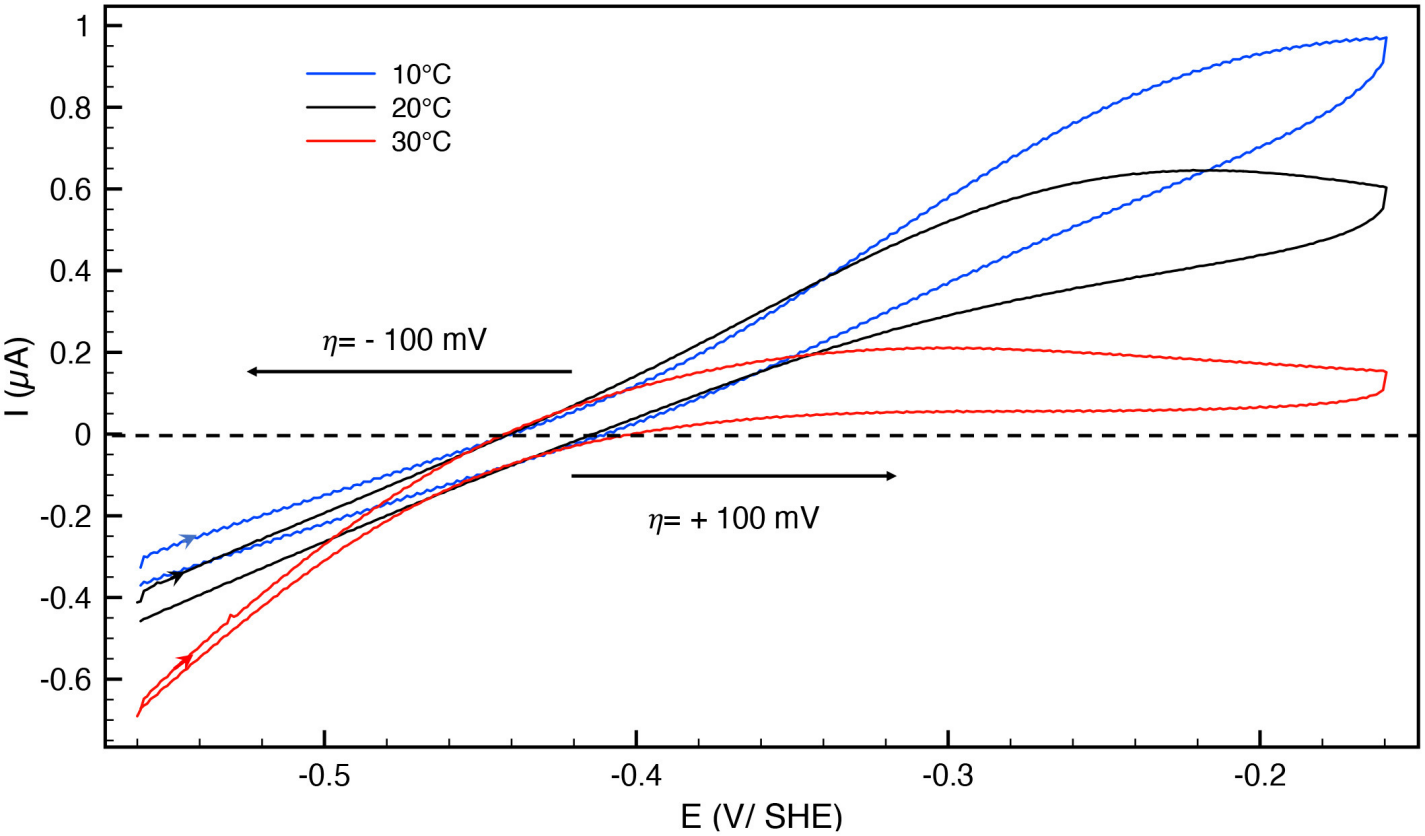
Cyclic voltammograms of anaerobically-purified Hnd hydrogenase adsorbed on PGE electrode as a function of temperature; blue: 10°C, black: 20°C, red: 30°C. Scan rate: 20 mV/s, 1 bar H_2_, phosphate buffer pH 7, ω = 3,000 rpm.

The catalytic bias [or catalytic preference (Sensi et al., 2017; Del Barrio et al., 2018b; Fourmond et al., 2019)] is defined as the propensity of a catalyst to catalyze a reaction faster in one direction than in the other. For the same positive and negative overpotential, the activity of the enzyme (i.e., the absolute value of the current in electrochemical experiment) must be compared. The catalytic bias is mainly the function of the rate limiting step of the catalyzed reaction. When inactivation happens, it influences the bias. As shown in Figure 1 anaerobically-purified Hnd is biased toward hydrogen production because it inactivates while oxidizing H_2_. Because inactivation and catalytic rate constants vary with temperature, the bias can be modulated by changing the temperature, as illustrated in Figure 2. While anaerobically-purified Hnd is biased toward proton reduction at 30°C, the opposite is true at 10°C (see data in Table 2 for a chosen overpotential of η = ± 100 mV, the current of the forward scan was considered). The same behavior was obtained for aerobically- and anaerobically-purified Hnd (Figure 2, Supplementary Figure 1 and Table 2). It should be noted that the potential range is not the same in Figure 1 (−0.56 to +0.04 V) and in Figure 2 (−0.56 to −0.16 V); thus the second inactivation that happened around −0.15 V is not visible in Figure 2.

**Table 2:** Catalytic bias data extracted from Figure 2 and Supplementary Figure 1, for an overpotential η = ±100 mV.

**Temperature**	**I (η = 100 mV) (in μA)**	**I (η = −100 mV) (in μA)**	**Bias**	
			**I (η = 100 mV)/**|****	**I (η = −100 mV)**|****
			**anaerobic**	**aerobic**
30°C	0.20	−0.45	0.44	0.21
20°C	0.50	−0.30	1.67	1.25
10°C	0.55	−0.20	2.75	1.93

#### 3.2.2. Reductive Inactivation

**Figure 3:**
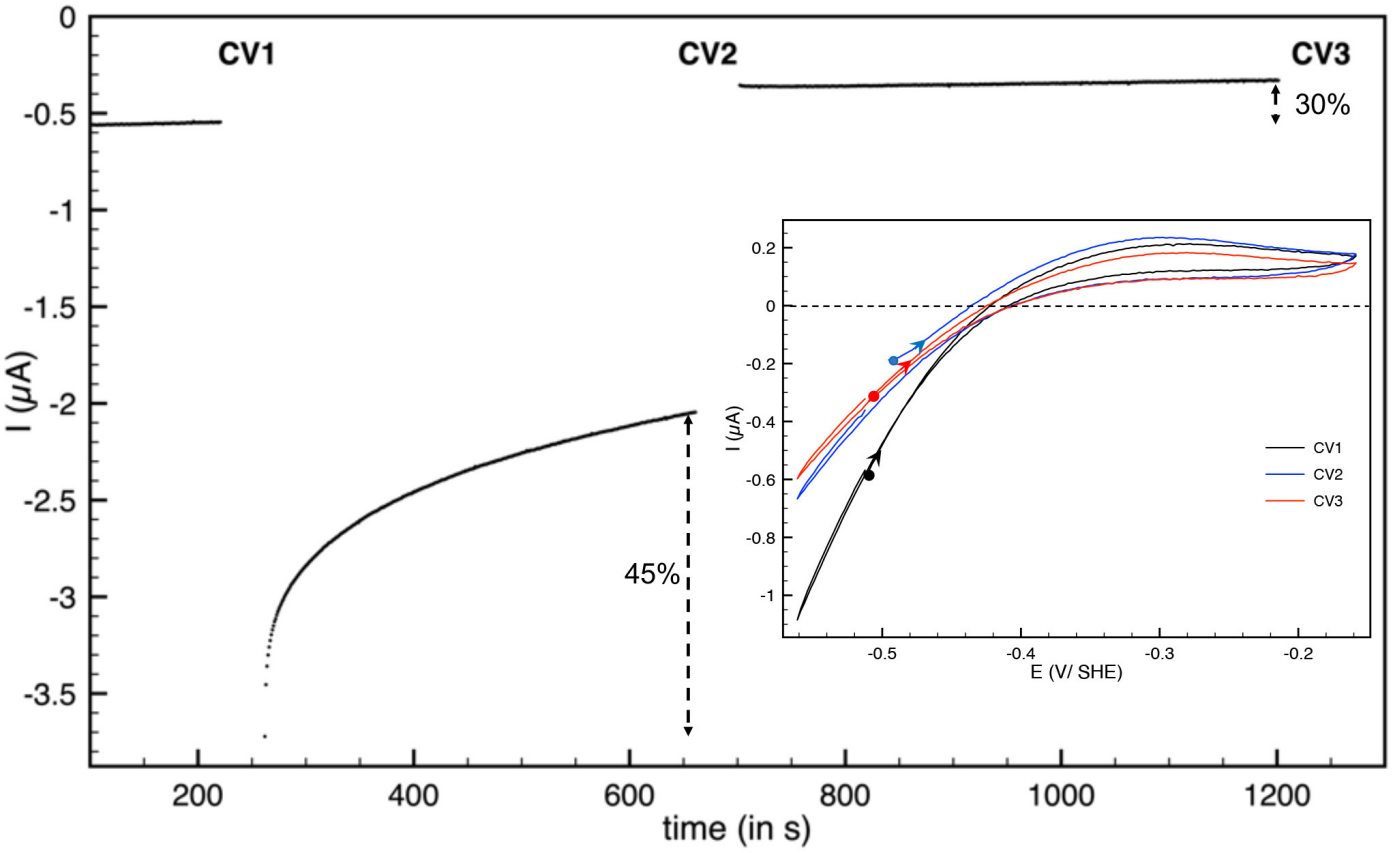
Reductive inactivation of aerobically-purified Hnd hydrogenase adsorbed on PGE electrode. Main: chronoamperogram, *E* = −510 mV vs. SHE for *t* < 250 s and *t* > 700 s and *E* = −760 mV vs. SHE for 250 < *t* < 700 s. Insert: cyclic voltammograms recorded after the first potential step (CV1, black line), after the second step (CV2, blue line), and after the last step (CV3, red line). The background current was subtracted. *T* = 30°C, 1 bar H_2_, phosphate buffer pH 7, ω = 3,000 rpm.

Hnd hydrogenase also inactivates at low electrode potential. We used the procedure described previously to study this inactivation (Hajj et al., 2014): a chronoamperogram was recorded while the electrode potential was changed in a 3-step-sequence (E_1_-E_2_-E_1_). E_1_ and E_2_ values were chosen, such as no activation nor inactivation occurs at electrode-potential E_1_, and such as detectable reductive inactivation takes place at electrode potential E_2_. After each step, a cyclic voltammogram (CV) was recorded. The CVs were started at −0.5 V vs. SHE and first scan to high potential. As shown in Figure 3, during the step at *E* = −0.76 V vs. SHE, 45% of the reduction current (i.e., enzyme activity) is lost, while the loss is only 30% after the third step (percentage of the reduction current loss between the end of the first step and the third step) indicating that inactivation is mostly irreversible. No reactivation was detected during this third step but fast inactivation must take place during CV2. The shape of the cyclic voltammogram after low potential inactivation is similar to the initial, thus the catalytic properties (including the bias) were not much affected by the low potential step. The same behavior was obtained for aerobically-purified and anaerobically-purified Hnd (Figure 3 and Supplementary Figure 2).

#### 3.2.3. Determination of the K_m_ for H_2_

We measured the Michaelis constants (k_m_) by examining how the steady-state H_2_ oxidation current depends on H_2_ concentration as described in Fourmond et al. (2013). The value indicated in Table 3 for Hnd hydrogenase was determined from three independent experiments. The same value was obtained for Hnd purified either under aerobic or anaerobic conditions.

**Table 3:** Comparison of the kinetic parameters determined by electrochemistry of different FeFe hydrogenases, including Hnd.

**Enzyme**	**K_m_ (H_**2**_) (in bar)**	**k_i_^CO^ (in mM CO^-1^·s^-1^)**	**k_a_^CO^ (in s^-1^)**	**K_i_^CO^ (in mM CO)**	**k_eff_^O_**2**_^ (in mM O_**2**_^-1^·s^-1^)**	**k_in_^O_**2**_^ (in mM O_**2**_^-1^·s^-1^)**
*C.a*. HydA	0.8 (30°C)^a^	8 (30°C)^b^	0.03 (30°C)^c^	3.75 × 10^−3^	0.05 (20°C)^b^; 0.077 (12°C)^d^	0.9 (20°C)^b^; 1.1 (12°C)^d^
*C.r*. HydA1	0.6 (30°C)^a^	80 (30°C)^b^	0.015 (30°C)^c^	1.9 × 10^−4^	1.02 (12°C)^d^	2.5 (12°C)^d^
*D.d*. HydAB	0.27 (30°C)^e^	1000 (30°C)^e^	0.03 (30°C)^e^	3 × 10^−5^	n.d.	40 (30°C)^e^
*M.e*. HydA	0.58 (5°C)	2 (20°C)^b^	0.003 (20°C)^b^	4.5 × 10^−3^	0.075 (20°C)^b^	0.25 (20°C)^b^
*A.w*. HDCR	0.24 (30°C)^f^	930 (30°C)^f^	0.02 (30°C)^f^	2.1 × 10^−5^	2.5 (30°C)^f^	6.5 (30°C)^f^
*D.f*. HndABCD	0.55 ± 0.15 (30°C)^g^	1,000 ± 340 (30°C)^g^	0.05± 0.02 (30°C)^g^	5 × 10^−5^	n. d.	2.4 ± 1.6 (12°C)^g^

a Fourmond et al. (2013);

b Caserta et al. (2018);

c Baffert et al. (2011),

d Orain et al. (2015),

e Liebgott et al. (2010);

f Ceccaldi et al. (2017);

g *This study, the values are the average of three independent experiments both for Hnd purified under aerobic or under anaerobic conditions. The k_i_^CO^ values are corrected for the effect of hydrogen protection (Liebgott et al., 2010). K_i_^CO^ = k_a_^CO^/k_i_^CO^. C.a., Clostridium acetobutylicum; C.r., Chlamydomonas reinhardtii; D.d., Desulfovibrio desulfuricans; M.e., Megasphaera elsdenii; A.w., Acetobacterium woodii; D.f., Desulfovibrio fructosovorans*.

#### 3.2.4. CO and O_2_ Inactivation

As the other FeFe hydrogenases, Hnd is inactivated in the presence of gas inhibitors, such as CO and O_2_. We determined the kinetic constants of these inhibitions and compared their values with those determined for other FeFe hydrogenases previously characterized electrochemically.

##### 3.2.4.1. CO Inactivation

**Figure 4:**
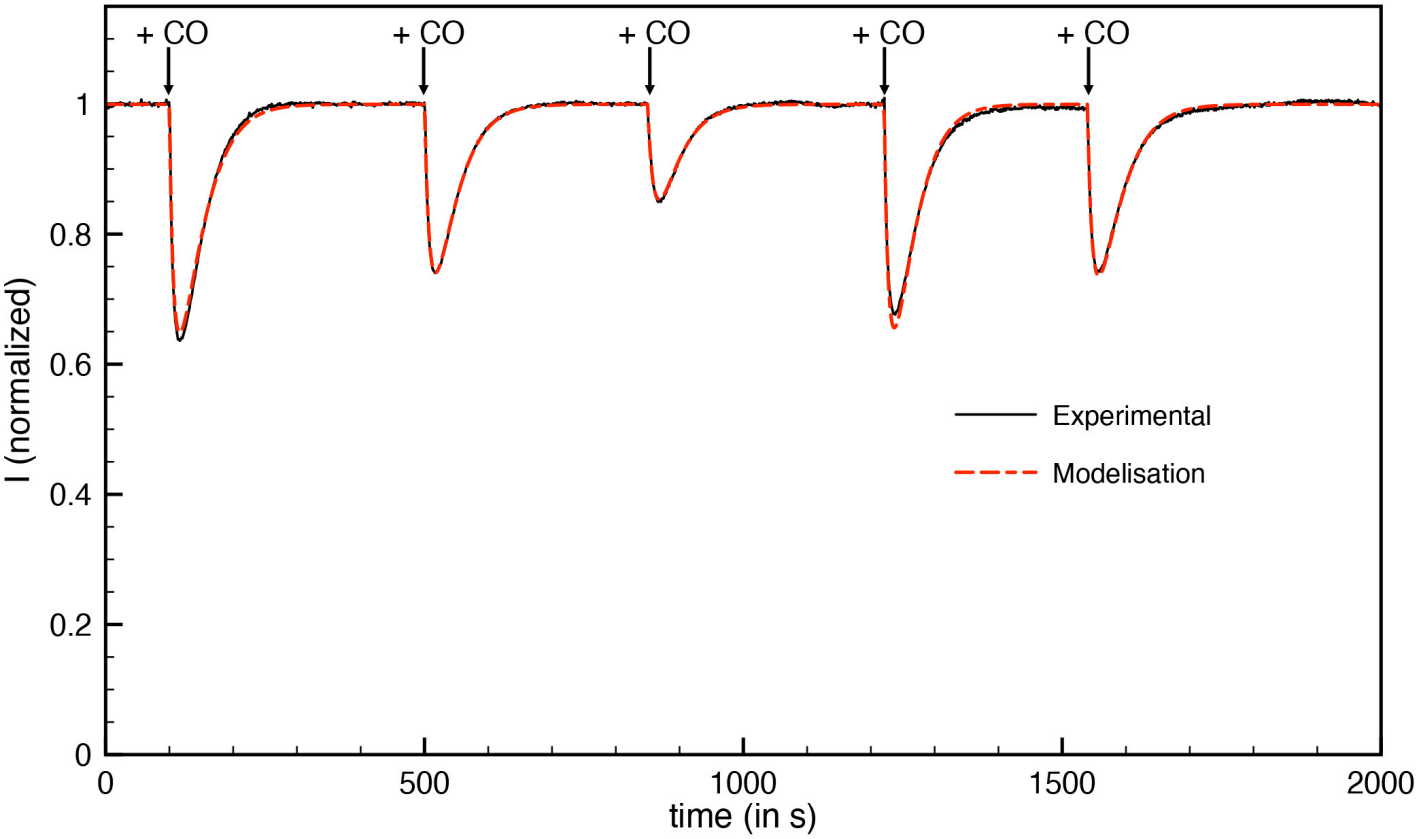
Inactivation by CO of aerobically-purified Hnd hydrogenase adsorbed on PGE electrode. The black line corresponds to experimental data and the dashed red line corresponds to best fit of the model in Equation (1). [CO] = 288, 192, 96, 288, and 192 nM injected at respectively *t* = 100, 500, 850, 1,220, and 1,540 s; *E* = −360 mV vs. SHE, *T* = 30°C, 1 bar H_2_, phosphate buffer pH 7, ω = 3,000 rpm.

CO inactivation was studied using the method described previously (Baffert et al., 2011): small aliquots of CO solution (1% CO and 99% Argon) were added while the current was measured at a constant electrode potential (Figure 4). We determined the kinetic rate constants of CO binding k_i_^CO^ and CO release k_a_^CO^ by fitting the model in Equation (1). The inactivation rate constants (k_i_^CO^) were corrected for hydrogen protection (Liebgott et al., 2010). The values are summarized in Table 3, leading to an inhibition constant K_i_^CO^ of 50 nM at 30°C.

##### 3.2.4.2. O_2_ Inactivation

**Figure 5:**
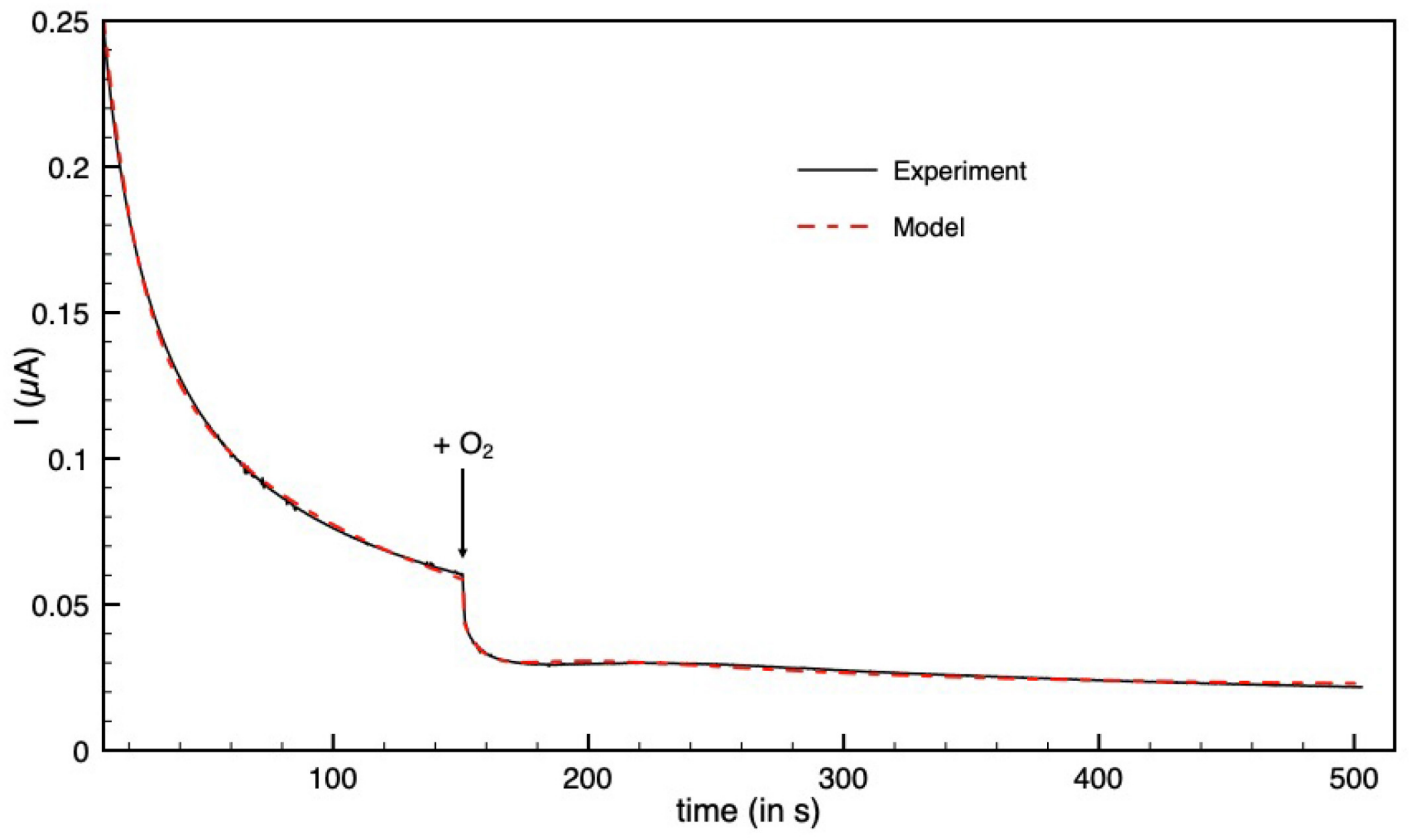
Inactivation by O_2_ of aerobically-purified Hnd hydrogenase adsorbed on PGE electrode. The black line corresponds to experimental data and the dashed red line is the best fit of the model in Equation (2). [O_2_] = 48 μM injected at *t* = 150 s. *E* = +40 mV vs. SHE, *T* = 12°C, 1 bar H_2_, phosphate buffer pH 7, ω = 3,000 rpm.

Figure 5 shows a chronoamperogram during which an aliquot of O_2_-saturated solution is injected at *t* = 150 s, then O_2_ is flushed away and its concentration in the electrochemical cell decreases exponentially over time. Before the injection, the decrease in current is due to the oxidative anaerobic inactivation described in section 3.2.1.1. When O_2_ is added, the current drops. A small part of this drop is due to direct reduction of O_2_ at the electrode, resulting in a negative current. This contribution was taken into account in the modeling. When the dioxygen is flushed out from the solution, a small reactivation could be detected. We used the model of Equation 2 to obtain the modelized curve in Figure 5. Because the initial oxidative anaerobic inactivation is biphasic, the model includes two inactive species of the enzyme (named inactive1 and inactive2) formed during this inactivation. Then a “dead-end” species is formed by O_2_ inhibition with a rate that depends on dioxygen concentration. We also tested the model with partly reversible O_2_ inactivation, such as described in ref (Orain et al., 2015) but it cannot be fitted to the experimental data. A kinetic constant k_in_^O_2_^ of 2.4 mM ${\text{O}}_{2}^{-1}\cdot$ s^−1^ was obtained with the modelization (Table 3).

Similar values for CO and O_2_ inhibition kinetic constants were obtained for Hnd purified either under aerobic or anaerobic conditions.

## 4. Discussion

The conditions of purification have little influence on the enzymatic properties of the Hnd hydrogenase (K_m_ for methyl-viologen, K_m_ for H_2_, optimal pH, and temperature). The purification conditions slightly influence the activity of the enzyme. The difference in activity between anaerobically- and aerobically-purified Hnd is greater with physiological partners using the electron bifurcation mechanism than with artificial redox partner (MV) (Table 1). These results suggest that aerobic purification could partly damage the enzyme, disrupting the complex. This would explain the decrease in H_2_-oxidation activity and the higher impact on the electron bifurcation reaction.

The catalytic and inactivation rates, i.e., the shape of the cyclic voltammogram in Figure 1, depend on the conditions of the purification of Hnd (aerobic vs. anaerobic). One additional inactivation process occurs at lower potential for the anaerobically purified Hnd, and this inactivation disappears after 1 day of storage (see insert of Figure 1). This indicates the presence of two forms of the enzyme that can interconvert. The presence of the two forms and their interconversion hinder a full characterization of the oxidative inactivation. However, the process appears to be biphasic (see Figure 5 before the addition of dioxygen) with the formation of two different inactive species (named inactive1 and inactive2 in Equation 2). The molecular difference between the two forms is still unknown, and could be due to a small change in the environment of the active site, as well as a conformational change. Further characterization of the interconversion and the oxidative inactivation is in progress in our laboratory.

Hnd is inactivated under very mild oxidative conditions compared to standard prototypic FeFe hydrogenases (Del Barrio et al., 2018a), especially when Hnd is purified under anaerobic conditions. This low potential oxidative inactivation was observed for two other hydrogenases: CpIII from *Clostridium pasteurianum* (Artz et al., 2019) and CbA5H from *Clostridium beijerinckii* SM10 (Corrigan et al., 2020; Happe et al., unpublished). This property was attributed in CpIII hydrogenase to the lack of polar residues in the vicinity of the H-cluster inducing a low dielectric permittivity (ε). However, by looking at the amino acids in the vicinity of the H-cluster (see Supplementary Table 4), this conclusion is not valid for CbA5H hydrogenase and even less for Hnd hydrogenase. Furthermore, considering Hnd, the kinetics of the oxidative inactivation and thus the potential at which it occurs depends on the conditions of purification while the amino acid composition is unchanged. This suggests that small changes around the H-cluster tune the rates of the oxidative inactivation process.

The bias depends on the rate of catalysis in either direction considered, but also on the rate of the oxidative inactivation. The two depend on temperature, thus the bias could be influenced by the temperature. As shown in Table 2, the catalytic bias can change within only 10°C, from being toward H_2_-production (at 30°C) to being toward H_2_-oxidation (at 20°C). Such a change has not been reported before with any enzyme.

In addition to oxidative inactivation, Hnd also inactivates under reducing condition (Figure 3). The shape of the cyclic voltammogram after low potential inactivation is similar (only small changes are visible) to the initial, unlike the case of HydA1 from *C. reinhardtii* (Hajj et al., 2014). In the case of HydA1 from *C. reinhardtii*, the change in the shape of the cyclic voltammogram was attributed to the formation of a form of the enzyme with catalytic activity different from that of the just purified enzyme. In the case of Hnd, the species formed under very reductive conditions is fully inactive or the reactivation is so fast that only the active enzyme is present at the beginning of the scan. Further investigations are needed to fully understand this process and to understand the differences with the other FeFe hydrogenases.

Table 3 compares the kinetic constants and inhibition constant by CO and O_2_ of various FeFe hydrogenases, including Hnd. With a value of 0.55 bar, the K_m_ for H_2_ is similar to that observed for the other FeFe hydrogenases. It should be noted that our set-up does not allow for a pressure of H_2_ >1 atm., which implies that the large value of K_m_ is only measured with low accuracy. CO is a competitive inhibitor of H_2_ oxidation by FeFe hydrogenases and H_2_ has a protective effect even if the K_m_ for H_2_ is high, so we chose to consider the true inhibition constant and not the apparent inhibition constant (Liebgott et al., 2010). While the reactivation kinetic constant (k_a_) does not differ much from one FeFe hydrogenase to the other (around 0.02 s^−1^), the inhibition kinetic constant (k_i_) is very dependent on which hydrogenase is considered (Table 3 and Caserta et al., 2018). The value obtained for Hnd is similar to that observed for the HydAB hydrogenase from another *Desulfovibrio* bacterium (*D. desulfuricans*) (Goldet et al., 2009; Liebgott et al., 2010) but also to that of HDCR from *Acetobacterium woodii* (Ceccaldi et al., 2017). The low inhibition constant (K_i_) of these three hydrogenases is probably not due to their multimeric composition but rather to the CO diffusion kinetics to the H-cluster. However, the fast diffusion of CO into these three hydrogenases could be attributed to a higher flexibility needed for the complex formation as proposed by Marsh and Teichmann (2014).

Hnd retains activity even when it is purified under aerobic conditions because it forms an O_2_-protected state named H_ox_^inact^ (Kpebe et al., 2018). Recent studies showed that in the H_ox_^inact^ state, a sulfur atom binds the H-cluster: it is either exogenous sulfur (present in the culture media) in the case of “standard” hydrogenases or the sulfur atom of a cysteine in the case of *Clostridium beijerinckii* (Rodríguez-Maciá et al., 2018; Corrigan et al., 2020 and Happe, communication at the 2019 Hydrogenase conference). We recorded CVs in absence and in presence of Na_2_S (Supplementary Figure 6) and we observed the same effect as that described with *D. desulfuricans* while Na_2_S has no effect on CbA5H FeFe-hydrogenase from *Clostridium beijerinckii* (Corrigan et al., 2020). Once it is activated under reducing conditions, it becomes sensitive to dioxygen inhibition as shown in Figure 5, which has been already observed for HydAB from *D. desulfuricans* (Roseboom et al., 2006; Rodríguez-Maciá et al., 2018). Unlike the FeFe hydrogenase CbA5H from *Clostridium beijerinckii* (Morra et al., 2016; Corrigan et al., 2020), Hnd is not converted from the active state back to the H_ox_^inact^ state when exposed to O_2_. Many FeFe hydrogenases can not form H_ox_^inact^ state and are inhibited by O_2_, either irreversibly or partially reversibly when O_2_ is flushed away. In the later case, the overall O_2_-sensitivity is defined by the effective inhibition rate constant k_eff_^O_2_^ (Table 3) (Caserta et al., 2018). Hnd data are better fitted to the model in Equation 2 with the inhibition by O_2_ being irreversible, and only the kinetic constant k_in_^O_2_^ can be determined. It could be directly compared to k_eff_^O_2_^. The sensitivity to O_2_ of Hnd is much higher than that of HydA1 from *C. acetobutylicum* and HydA from *M. elsdenii* but similar to that of HydA1 from *C. reinhardtii* and HDCR from *Acetobacterium woodii*. *Desulfovibrio* bacteria can face transient exposure to dioxygen under physiological conditions and indeed possess oxygen reductases (Dolla et al., 2006; Schoeffler et al., 2019). Furthermore, different enzymes from *Desulfovibrio* species were shown to resist exposure to O_2_ [pyruvate:ferredoxin oxidoreductase (Vita et al., 2008) and CO-dehydrogenase (Merrouch et al., 2015)]. The reversible oxidative inactivation of Hnd could be another mechanism for O_2_-protection, under physiological conditions.

## 5. Conclusion

Here were reported the full enzymatic characterization, using biochemical and electrochemical methods, of the electron-bifurcating hydrogenase Hnd from *D. fructosovorans* purified either under aerobic or anaerobic conditions. While usually the conditions of purification are not considered (or the purification is only possible under one condition), we show that the conditions of purification could influence the properties of the enzyme: the presence or the absence of air during purification leads to two different forms of the enzyme that can interconvert. These two forms show differences in the catalytic properties, mainly regarding the oxidative inactivation. The anaerobic oxidative inactivation of one of the forms of the enzyme occurs at relatively low potential compared with other characterized hydrogenases, a behavior already observed with two other FeFe hydrogenases: CbA5H from *Clostridium beijerinckii* (Corrigan et al., 2020) and CpIII from *Clostridium pasteurianum* (Artz et al., 2019). The other catalytic properties (K_m_, CO, and O_2_ inhibition constant) do not depend on the conditions of purification and are comparable to those of prototypic FeFe hydrogenases already characterized. This indicates that the presence of additional subunits in Hnd complex has little effect on the catalytic and inactivation properties of the hydrogenase. The characterization of the isolated hydrogenase subunit HndD is in progress to verify this hypothesis.

### 5.1. Equations

(1) $$\begin{array}{l} \text{Active}\overset{k_{\text{i}}^{\text{CO}}\times \;[\text{CO}]}{\underset{k_{\text{a}}^{\text{CO}}}{⇌}}\text{Inactive} \end{array}$$

(2) $$\begin{array}{l} \text{Inactive2}\overset{k_{2_{1}}}{\underset{k_{1_{2}}}{⇌}}\text{Inactive1}\overset{k_{1_{A}}}{\underset{k_{A_{1}}}{⇌}}\text{Active}\overset{k_{\text{i}}^{{\text{O}}_{2}}\times [{\text{O}}_{2}]}{\to }\text{Dead end} \end{array}$$

## Data Availability Statement

The original contributions presented in the study are included in the article/Supplementary Material, further inquiries can be directed to the corresponding author/s.

## Author Contributions

AJ-B, MBe, NP, AK, and CB purified the proteins and performed experiments. CF, VF, and CB analyzed the data. CL, MBr, and CB instigated the research. All the authors wrote the manuscript.

## Conflict of Interest

The authors declare that the research was conducted in the absence of any commercial or financial relationships that could be construed as a potential conflict of interest.

## Abbreviations

FMN: flavin mononucleotide
NAD: nicotinamide adenine dinucleotide
NADP: nicotinamide adenine dinucleotide phosphate
*bis*PGD: *bis* pyranopterin guanosine dinucleotide
Fd: ferredoxin
SHE: standard hydrogen electrode.
