# Supplementary material for: Electrochemical Characterization of a Complex FeFe Hydrogenase, the Electron-Bifurcating Hnd From Desulfovibrio fructosovorans
Source: Front Chem. 2021 Jan 8;8:573305. doi: 10.3389/fchem.2020.573305 (PMC7820892; doi:10.3389/fchem.2020.573305)
Supplement: Supplementary file 1 [file Table_1.DOCX]

Supplementary Material

S1: Cyclic voltammograms of aerobically-purified Hnd hydrogenase adsorbed on PGE electrode as a function of temperature; blue: 10°C, black: 20°C, red: 30°C. Scan rate: 20mV/s, 1 bar H_2_, phosphate buffer pH 7, 𝜔= 3000 rpm

S2: Reductive inactivation of anaerobically-purified and aerobically-purified Hnd hydrogenase adsorbed on PGE electrode. Chronoamperogram, E= -510 mV *vs* SHE for t<100 s and t>150s and E= -760 mV *vs* SHE for 100<t<150 s. The background current was subtracted. T=30◦C, 1 bar H2, phosphate buffer pH 7, ω= 3000 rpm.

S3: Modelization parameters for O_2_-inhibition (Figure 5)

Direct O_2_ reduction at the electrode current= -3x10^-7^ A

[O_2_]_0_ = 0.048 mM

𝜏 O_2_= 15.6 s (rate of O_2_ flushing away from the electrochemical cell)

k_a1_ = 0.0248 s^-1^

k_1a_ = 0.0197 s^-1^

k_1_2_ = 0.0107 s^-1^

k_2_1_ = 0.0024 s^-1^

S4: Amino-acids in the vicinity of the H-cluster for several hydrogenases

|  | CpI | CpII | CpIII | HydA1 Cr | HydA Ca | HydAB  Dd | Cb5AH | HydABC  Tm | Hnd  Df |
| --- | --- | --- | --- | --- | --- | --- | --- | --- | --- |
|  | P354 | P | P | P | P | P | P | P | P |
|  | M353 | **T** | *G* | M | M | M | M | M | M |
|  | **K358** | **K** | **K** | **K** | **K** | **K** | **K** | **K** | **K** |
|  | **C299** | **C** | **C** | **C** | **C** | **C** | **C** | **C** | **C** |
|  | **Q325** | I | M | **Q** | **Q** | V | M | **Q** | **Q** |
|  | **S323** | **S** | **S** | **S** | **S** | **S** | **S** | **S** | **S** |
|  | P324 | P | P | P | P | P | P | P | P |
|  | I268 | **T** | V | **T** | I | **T** | **T** | V | **T** |
|  | A230 | **S** | A | A | A | A | A | A | A |
|  | P231 | P | P | P | P | P | P | P | P |
|  | **S232** | **S** | A | A | **S** | A | A | A | A |
|  | M497 | M | M | M | M | M | M | M | M |
|  | V424 | V | V | V | V | V | V | V | V |
|  | F417 | F | ***Y*** | F | F | F | F | F | F |
|  | G418 | G | *A* | G | G | G | G | G | G |
|  | **N269** | **T** | A | L | **N** | **E** | **S** | **S** | **D** |
| **P, NC** | 5 | 7 | 3 | 4 | 5 | 3 | 4 | 4 | 4 |
| **P, C** | 1 | 1 | 1 | 1 | 1 | 2 | 1 | 1 | 2 |
| NP | 10 | 8 | 12 | 11 | 10 | 11 | 11 | 11 | 10 |
| P | 6 | 6 | 4 | 5 | 6 | 5 | 5 | 5 | 6 |

In bold: polar residues; P: polar residues, NC: non-charged residues, C: charged residues, NP: non-polar residues.

CpI, CpII and CpIII from *Clostridium pasteurianum*, HydA1 from *Chlamydomonas reinhardtii,* HydA from *Clostridium acetobutylicum*, HydAB hydrogenase from *Desulfovbrio desulfuricans*, Cb5AH from *Clostridium beijerinckii* SM10, HydABC from *Thermotoga maritima*, HndABCD from *Desulfovibrio fructosovorans*.

S5: Six successive cyclic voltammograms of aerobically-purified Hnd hydrogenase adsorbed on PGE electrode. Color code: from blue (scan 1) to red (scan 6). Temperature= 30°C, scan rate: 20mV/s, 1 bar H_2_, phosphate buffer pH 7, 𝜔= 3000 rpm.

S6: Cyclic voltammograms of aerobically-purified Hnd hydrogenase adsorbed on PGE electrode without (black line) and with 1mM Na_2_S (red line). Scan rate: 20mV/s, 1 bar H_2_, phosphate buffer pH 7, 𝜔= 3000 rpm, Temperature= 5°C.

S7: Specific activity and biochemical and catalytic properties of different enzyme preparation.

| Purification conditions | Specific activity (MV) H_2_ oxidation (in U/mg of enzyme) | Km (H_2_) (in bar) at 30°C | K_i_^CO^ (in mM CO) | Km (MV) (in mM) |
| --- | --- | --- | --- | --- |
| Aerobic | 475 | 0.55 | 5 10^-5^ | 13 |
| Aerobic | 2500 |  | 3.9 10^-5^ | 15 |
| Aerobic | 430 | 0.54 | 3.2 10^-5^ |  |
| Aerobic | 500 | 0.72 |  | 20 |
| Anaerobic | 745 | 0.62 | 4.7 10^-5^ |  |
| Anaerobic | 100 | 0.48 |  |  |
